# Isothermal Amplification Technology for Disease Diagnosis

**DOI:** 10.3390/bios12090677

**Published:** 2022-08-24

**Authors:** Poramin Boonbanjong, Kiatnida Treerattrakoon, Wassa Waiwinya, Piyawat Pitikultham, Deanpen Japrung

**Affiliations:** 1 National Nanotechnology Center, National Science and Technology Development Agency, Thailand Science Park, Pathumthani 12120, Thailand; 2 Program in Translational Medicine, Faculty of Medicine Ramathibodi Hospital, Mahidol University, Bangkok 10400, Thailand; 3 Department of Pure and Applied Chemistry, Technology and Innovation Centre, University of Strathclyde, Glasgow G1 1RD, UK; 4 Multidisciplinary Program of Medical Microbiology, Graduate School, Chulalongkorn University, Bangkok 10330, Thailand; 5 CAS Key Laboratory of Nanosystem and Hierarchical Fabrication, CAS Center for Excellence in Nanoscience, National Center for Nanoscience and Technology, Beijing 100190, China; 6 School of Nanoscience and Technology, University of Chinese Academy of Sciences, Beijing 100049, China

**Keywords:** isothermal amplification, nucleic acid amplification technology, loop-mediated isothermal amplification, rolling circle amplification, CRISPR

## Abstract

Isothermal amplification (IA) is a nucleic acid amplification technology (NAAT) that has contributed significantly to the healthcare system. The combination of NAAT with a suitable detection platform resulted in higher sensitivity, specificity, and rapid disease diagnosis. Traditional NAAT, such as polymerase chain reaction (PCR), is widely applied in the general healthcare system but is rarely accessed in resource-limited hospitals. Some IA methods provide a rapid, sensitive, specific, and simple method for disease diagnosis. However, not all IA techniques have been regularly used in clinical applications because different biomarkers and sample types affect either the enzyme in the IA system or sample preparation. This review focuses on the application of some IA techniques that have been applied in the medical field and have the potential for use at points of care.

## 1. Introduction

**Figure 1:**
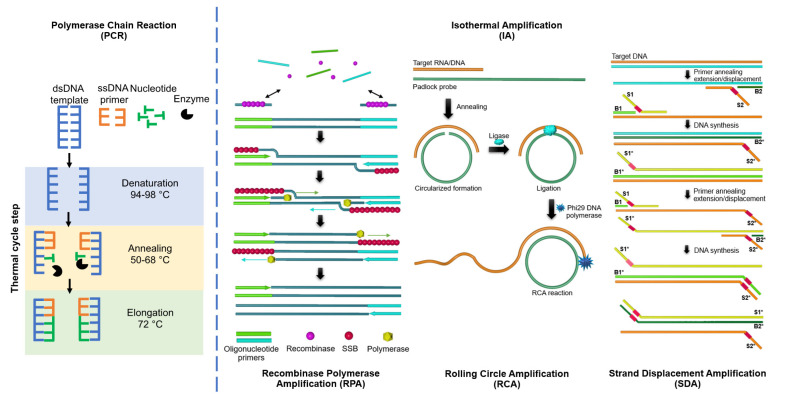
Schematic overview of polymerase chain reaction (PCR) and some isothermal amplification (IA) systems: recombinase polymerase amplification (RPA), rolling circle amplification (RCA), and strand displacement amplification (SDA).

Isothermal amplification (IA) is a technique for amplifying nucleotide chains using a simple temperature controller, and it is potentially used for point-of-care testing in fields or general laboratories. The major difference between IA and polymerase chain amplification (PCR) is that IA is performed at a constant temperature, whereas PCR requires a temperature-changing system. Another important difference between IA and PCR is that polymerase enzymes normally extend primers on a single strand of the dsDNA template during PCR. In contrast, some IA systems have a special polymerase enzyme that can directly extend primers on a double strand of dsDNA during the amplification process. In addition, some IA techniques, such as rolling circle amplification (RCA), have the ability to use DNA/RNA targets as primers and designed ssDNA/RNA as a template, as shown in Figure 1.

There are three key components of the IA system: (1) nucleic acid templates, (2) the initial primer for complementary binding to the target nucleic acids, and (3) isothermal enzymes for nucleic acid amplification. Both PCR and IA require DNA templates, primers, nucleotide substrates, and amplification enzymes. PCR templates are mostly dsDNA, which require a thermal cycle step to denature, anneal, and elongate the template and amplify the final dsDNA products. The templates for the IA system can be ssDNA, dsDNA, and RNA. Moreover, in the padlock probe-based RCA, one of the IA systems, the short target RNA can subsequently be used as a primer for RCA reaction, thereby linearly amplifying the target sequence in a process that has been shown to be highly quantitative. Some IA systems, such as recombinase polymerase amplification (RPA) and strand displacement amplification (SDA), can use dsDNA as a template and require single stranded binding protein (SSB), recombinase enzyme, and isothermal polymerase enzyme for strand separation and elongation. The significant advantages of the IA system are the ability to amplify nucleic acids using a simple thermal controller system and the simple operation requiring only less skilled staff to perform the test. Therefore, the IA system can be potentially applied for onsite testing in remote hospitals and healthcare settings. This review focuses on the applications of current IA techniques, such as LAMP, RCA, and CRISPR-Cas systems in the medical field. 

## 2. Loop-Mediated Isothermal Amplification (LAMP) 

LAMP was first described in 2000 by Notomi et al. as a nucleic acid amplification technique with simplicity, high specificity, efficiency, and speed at a constant temperature [1] In principle, LAMP requires a polymerase enzyme with strand displacement activity, such as phi29 DNA polymerase or *Bst* DNA polymerase, and a set of four to six specific primers that recognize six to eight different regions of the target DNA. 

**Figure 2:**
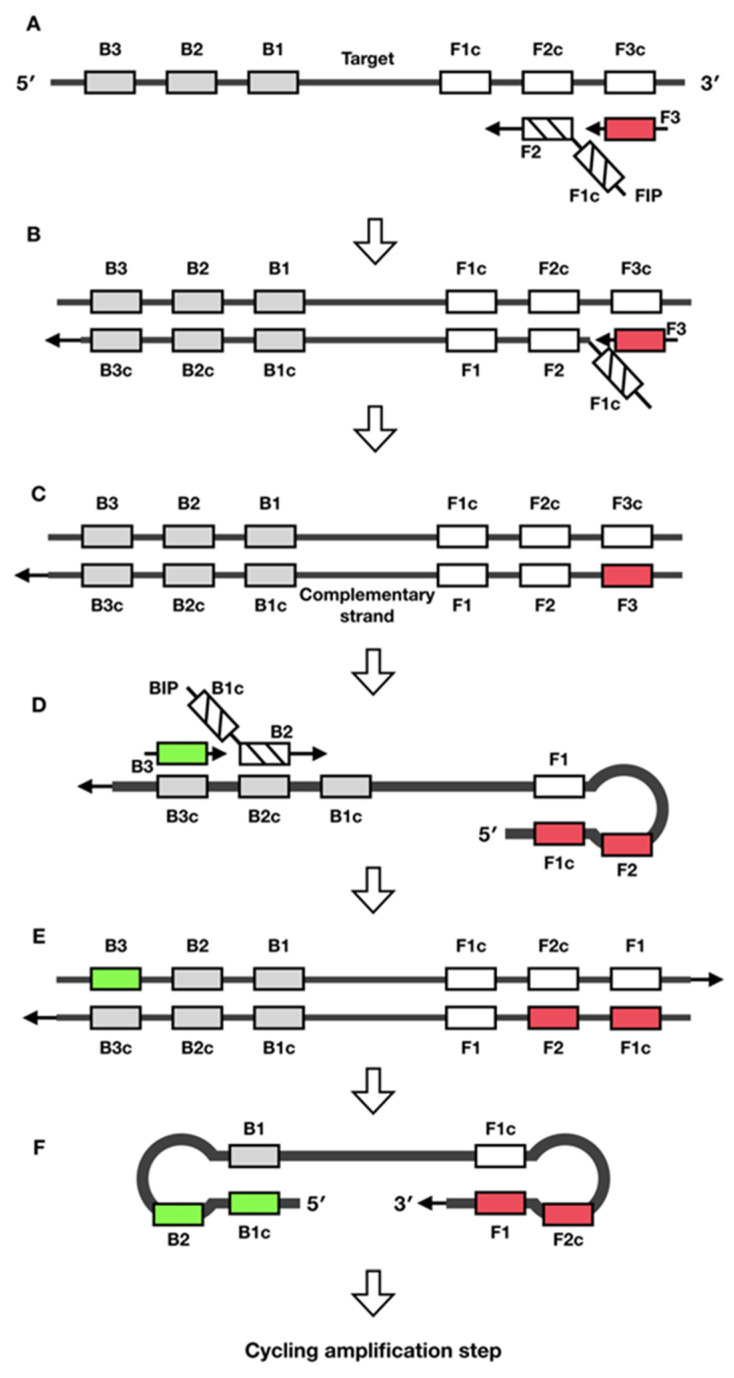
Schematic of LAMP mechanism (redrawn according to [1]).

The LAMP mechanism is illustrated in Figure 2. First, a forward inner primer (FIP) was hybridized to the F2c region of the target DNA and a complementary strand was synthesized (Figure 2A). Next, strand displacement was initiated after the outer primer F3 hybridized to the F3c region. The complementary strand was then released and served as a template for the backward inner primer (Figure 2B,C). Next, the backward inner primer (BIP) was hybridized, and strand synthesis was initiated. Subsequently, the strand was displaced, using the B3 primer (Figure 2D). Finally, complementary DNA strands were synthesized entirely, and dumbbell-shaped or stem-loop DNA was produced for the LAMP cycling amplification (Figure 2E,F).

The product of LAMP can be detected either by turbidity from magnesium pyrophosphate precipitation [2], gel electrophoresis, naked-eye, or intercalating DNA dyes, such as SYBR Green I [3]. LAMP is widely used as a screening tool for several diseases. LAMP coupled with a colorimetric DNA-based magnetogenosensor has been used to detect *Leptospira* DNA [4]. The total DNA from 172 bacterial strains, including pathogenic, intermediate, non-pathogenic Leptospira, and other pathogens, was isolated and amplified by LAMP. The amplified products were labeled with fluorescein isothiocyanate (FITC). Biotinylated capture, which acts as a DNA capture probe, was immobilized on the magnetic beads. Then, the LAMP products were hybridized with the DNA capture probe and 3,3′,5,5′-tetramethylbenzidine (TMB) was used as a substrate for colorimetric detection. The sensitivity of this biosensor platform for *Leptospira* was 200 fg/μL of target DNA, and the selectivity to pathogenic *Leptospira* was 100%.

LAMP has been used to detect *Mycobacterium tuberculosis*, *Mycobacterium avium*, and *Mycobacterium intracellulare* in sputum specimens [5]. The DNA of mycobacterial species can be detected with LAMP within 35–60 min after performing the reaction. The limit of detection for this assay is 5–50 copies of DNA per test. LAMP has also been used as a viral detection, screening, and diagnostic tool for diseases in the fishery industry. LAMP coupled with SYBR Green I was used for Koi Herpesvirus (KHV) detection in carp and koi fish [3]. First, DNA was extracted from fish and amplified with *Bst* DNA polymerase using the LAMP technique. The LAMP products can be detected visually by adding SYBR Green I and observing changes in the product color. This technique provides an easy and rapid diagnosis within 90 min and can be used outside the laboratory without special equipment.

Reverse transcription-LAMP (RT-LAMP) is an isothermal amplification-based RNA technique. Similar to RT-PCR, RNA was converted to cDNA using reverse transcription enzyme. Then, cDNA was further amplified using strand displacement DNA polymerase in one step. RT-LAMP was developed as a tool to detect Middle East respiratory syndrome coronavirus (MERS-CoV) with rapidity and high specificity [6,7]. Lee et al. developed the RT-LAMP technique that can detect MERS-CoV RNA with as few as 0.4 infectious viral genome copies/μL without cross-reaction to other acute respiratory disease viruses, such as influenza virus type A (H1N1, H3N2) and influenza virus type B. 

Furthermore, the detection of MERS-CoV using the RT-LAMP technique was developed by Shirato et al. RT-LAMP can detect MERS-CoV RNA within 1 h, with high sensitivity. The sensitivity of this technique was 3.4 copies of MERS-CoV RNA without cross-reactivity to other coronavirus strains and acute respiratory disease viruses. 

In 2019, LAMP was used as a detection platform for severe acute respiratory syndrome-related coronavirus (SARS-CoV) by Kim et al. They developed a simple LAMP coupled with a colorimetric assay and a multiplex LAMP targeting the open reading frame (ORF) and nucleocapsid (N) gene of SARS-CoV [8]. This technique detected the target RNA of SARS-CoV within 30 min. The sensitivity of this technique was 10^5^ copies per reaction for the ORF gene and 10^4^ copies per reaction for the N gene.

Recently, RT-LAMP has been used as a rapid tool to detect novel coronavirus (COVID-19) [9]. Briefly, RT-LAMP primers were designed using 23 different consensus sequences of COVID-19. Patient samples, including serum, saliva, nasopharyngeal swab, oropharyngeal swab, and urine, were spiked with COVID-19, Middle East respiratory syndrome (MERS), Betacoronavirus England-I (BtCoV), and murine hepatitis virus (MHV). The spiked patient samples were directly subjected to RT-LAMP using strand displacement DNA polymerase and *Bst* DNA polymerase. Next, SYBR Green I was used as a reporter dye and observed by the naked eye under UV conditions and gel electrophoresis. RT-LAMP can detect COVID-19 in mimicked patient samples within 30 min with high sensitivity and specificity. The limit of detection (LOD) of this technique was 1.02 fg. In addition to the advantages of the LAMP technique, it has some limitations, including false-positive results from self–primer or primer–primer hybridization. This technique also carries a high risk of contamination [10,11].

## 3. Rolling Circle Amplification (RCA)

RCA was first described in 1995 [12] as an isothermal enzymatic-based technique that uses a polymerase enzyme with strand displacement activity to amplify a target. This technique has emerged as a novel and potential tool in biomedical and clinical research. The RCA technique has several advantages, including simplicity, high sensitivity, and high selectivity. Moreover, unlike conventional polymerase chain reactions, amplification can be performed at a constant temperature. These advantages make RCA a potential tool for detecting microRNAs (miRNAs) and infectious pathogens, such as bacterial and viral nucleic acids.

In nature, the RCA reaction is a process of genomic replication, which can rapidly produce multiple copies of circular nucleic acid molecules, such as bacteriophage genomics and plasmids of viruses [12,13]. There are five essential components in the RCA reaction: (1) a short linear DNA/RNA strand that acts as a primer for the utilization of a circular template, (2) the circular template or padlock probe with 5′- phosphate group modification, (3) the specific DNA/RNA polymerase (e.g., Phi29 DNA polymerase), (4) the DNA/RNA ligase enzyme (e.g., T4 DNA ligase), and (5) deoxynucleotide triphosphate (dNTP), which is a monomer unit of RCA [14,15]. First, the RCA reaction can be initiated after hybridization between the linear primer and the linear padlock probe, which induces the formation of a circular structure. Ligase enzymes can covalently join both terminal ends of the circular template via the phosphate group of the DNA/RNA sugar backbone to form a closed-loop structure. Subsequently, DNA/RNA polymerase catalyzes polymerization from the 5′-to-3′ direction [16]. The RCA product is a long, single-stranded DNA concatemer containing repeated sequences complementary to padlock probe sequences [17]. In comparison with other nucleic acid amplification methods, the RCA reaction can be performed at low temperatures, approximately 23 °C to 60 °C [18]. Conventional polymerase chain reaction requires a wide range of temperature cycling controls. By discreet design of padlock probe sequences, versatile functions, such as detection, amplification, and cleavage regions, can be appropriately customized and controlled. Based on the RCA concept, an amplified signal can be generated under a single binding situation at low abundance [19,20]. The plurality product of the RCA can be integrated with other methods for exponential amplification. One example is hyperbranched RCA (HRCA) in which the introduction of a secondary primer to the RCA product can further expand the amplification [21] and nicking-enhanced RCA to catalyze the RCA product into a plurality of primers for the next cycle of RCA [22,23].

RCA products can be monitored and read using various technologies. Among these, gel electrophoresis is the conventional procedure for RCA product analysis [24]. However, one of the simplest methods is a fluorescence-based technique, such as RCA product binding fluorescence dye and complementary strand induced fluorescence response [25,26]. Furthermore, some applications have integrated RCA products with nanomaterials (e.g., AuNPs, AgNPs, quantum dots, and magnetic beads) to enhance signal detection and visualization [27,28,29]. For example, RCA hybridization with quantum dots and metal nanomaterials can provide a detectable electrochemical signal [27], DNA-intercalating dyes (e.g., SYBR Green) can exhibit a quantified fluorescence signal [30], and a combination with horseradish peroxidase (HRP) can induce a change in color, which can be observed by the naked eye [31]. Hence, the RCA technique has several advantages, including simplicity, high sensitivity, and high selectivity, making it a potential tool for versatile detection of infectious pathogens, bacteria, viral nucleic acids, and microRNAs (miRNA). 

In 2010, RCA was developed as a platform for double-stranded DNA (dsDNA) detection and was used to detect the genomic DNA of *Mycobacterium tuberculosis* by Schopf et al. DNA capture probes were immobilized on Sepharose beads. Subsequently, dsDNA was denatured and separated into two single strands before hybridization with capture and padlock probes. Ligation and circularization steps were then initiated, and the target DNA was amplified by RCA reaction using phi29 DNA polymerase. Following the reaction, the target DNA was labeled with two fluorescently labeled (FAM and Cy3) oligonucleotide probes for imaging. *M. tuberculosis* genomic DNA was detected using this method. Next, long- and double-stranded DNA were digested with restriction enzymes into small fragments. The DNA fragment was then denatured, and the RCA reaction was performed in the same manner as for dsDNA detection. The limit of detection of this platform was 4.25 fM of target dsDNA and 10,000 cfu/mL of *M. tuberculosis* [32].

Primer-generation RCA (PG-RCA), a novel RCA model, was developed to detect the food-borne pathogen *Listeria monocytogenase* [19]. First, the genomic DNA of *L. monocytogenes* was denatured and hybridized using a circular probe. Subsequently, a long-concatenated circular probe sequence was synthesized using vent (exo-) DNA polymerase through the L-RCA reaction. Next, multiple sites of the L-RCA product were hybridized using multiple circular probes and recognized by the nicking enzyme. Finally, the recognition sequences were cleaved by the nicking enzyme and the generated primer of the circular probe. Fluorescence intensity was monitored using SYBR Green I. The sensitivity of this technique for *L. monocytogenes* genomic DNA detection was 0.163 pg or approximately 60 molecules.

RCA has also been used for the detection of viral nucleic acid detection. In 2015, Hamidi et al. developed an RCA platform to detect H5N1 influenza viral RNA with high sensitivity using hyperbranched RCA (HRCA) combined with fluorometric detection [33]. In brief, a circular padlock probe (C-PLP) was synthesized by phi29 DNA polymerase and amplified by HRCA. During the HRCA reaction, SYBR Green I, a fluorescent reporter molecule, was added to double-stranded DNA. The sensitivity of this technique was as low as 9 fM. 

The RCA detection platform for severe acute respiratory syndrome-related coronavirus (SARS-CoV) was developed in 2005 by Wang et al. They developed a detection technique for single-stranded RNA of coronaviruses using HRCA [34]. The HRCA for SARS-CoV RNA was tested on the surface of a magnetic bead coated with oligo (dT) and in a reaction buffer, which is called the solid and liquid phases, respectively. Briefly, in the molecular model of the liquid phase, probe-specific SARS-CoV target RNA was ligated and circularized using DNA ligase. The circular probe was then amplified using *Bst* DNA polymerase at a constant temperature. In contrast, magnetic beads coated with oligo (dT), which binds mRNA, were used for the RCA reaction in the solid phase. The viral RNA-bound magnetic beads were then separated, and the RCA reaction was performed as a liquid phase. The advantage of this technique is its high sensitivity as a single copy of SARS-CoV can be detected. However, this technique still has some limitations, including the requirement of gel electrophoresis for result analysis and insufficient sample quantification. 

The high sensitivity and selectivity of RCA make it a feasible tool for detecting and quantifying miRNAs. Ligation RCA (L-RCA) was developed and used as a platform for miRNA detection by Hong et al. They developed an L-RCA coupled with graphene oxide (GO) and a fluorescently labeled peptide nucleic acid (F-PNA) probe as a reporter molecule [35]. First, the padlock probe (PLP) DNA was ligated to the target miRNA and circularized using phi29 DNA polymerase. Subsequently, circular DNA was used as a template, and F-PNA probes were annealed to multiple sites of the single-stranded RCA products. Finally, graphene oxide, which has a high affinity for single-stranded nucleic acids, was used for quantitative analysis by measuring the fluorescence intensity. The limit of detection of L-RCA coupled with GO and F-PNA was calculated to be as low as 0.4 pM of target miRNA. 

**Figure 3:**
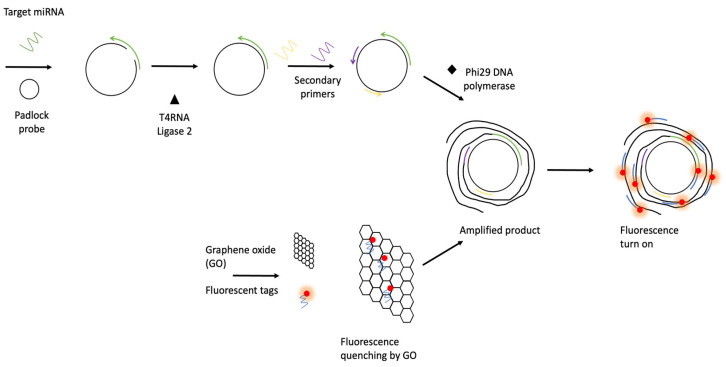
Schematic diagram of MPRCA-GO platform for miRNA detection (redrawn according to [36]).

Multiple primer-mediated rolling circle amplification (MPRCA) was developed to detect miRNAs [36]. Their study utilized a padlock probe comprising a target-binding region and two secondary primer-binding regions. When the 5′and 3′ ends of the padlock probe hybridized with the target miRNA, T4 RNA ligase 2 enzymes ligated the ends of the probe to form a circular template. The miRNA target acted as a primary primer, and two additional secondary primers acted as signal enhancers. Isothermal amplification was initiated with Phi29 DNA polymerase, resulting in long concatemers of the MPRCA products (Figure 3). 

**Figure 4:**
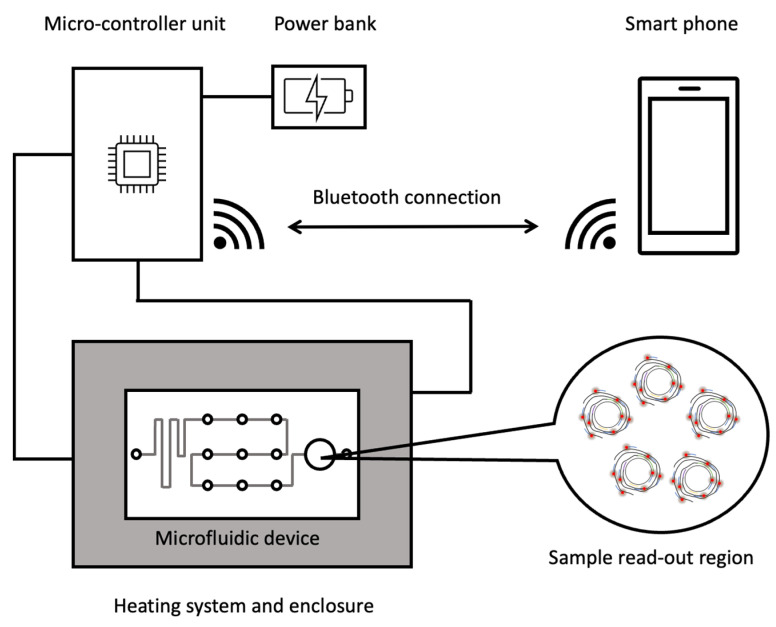
Schematic diagram of RCA and fluorescent-graphene based sensor-on-a-chip system, (redrawn according to [37]).

In addition, the developed MPRCA assay coupled with graphene oxide (GO) fluorescence-based sensing platform could detect lung cancer-associated miRNAs with high sensitivity in the femtomolar range. This technique can also be used to detect miRNAs in clinical specimens. Another study from the same group also integrated rolling circle amplification and a graphene-based sensor for miRNA detection into a microfluidic device with a mobile controller (Figure 4) [37]. The closed system of a microfluidic device with a mobile controller can reduce the risk of contamination and facilitate the potential use of this technique in point-of-care settings, without special laboratory equipment or specialized technicians.

## 4. Clustered Regularly Interspaced Short Palindromic Repeats (CRISPR) and CRISPR-Associated Protein (Cas) System 

CRISPR-Cas systems are groups of DNA sequences found in the genomes of prokaryotic organisms that serve as adaptive immunity against bacteriophages, viruses, and foreign genetic materials, such as plasmids [38]. Currently, polymerase chain reaction (PCR)-based techniques are the most commonly used standard methods for nucleic acid detection. However, the PCR technique has some limitations, including low sensitivity and requires sophisticated equipment and skilled technicians. A new approach for nucleic acid detection with simplicity, speed, and high sensitivity has been developed over the past decade to overcome these limitations. The CRISPR-Cas system has emerged as a novel approach for nucleic acid detection, with rapidity, high sensitivity, and specificity [39]. The CRISPR-Cas system can be classified into three groups based on the major characteristics of Cas effectors: Cas9, Cas13, and Cas 12 [40]. 

CRISPR-associated protein (Cas) 9 is an RNA-programmable DNA-targeting and editing tool that serves as an adaptive immune system in bacteria and archaea. The CRISPR-Cas9 system consists of two major components: Cas9 enzyme, an RNA-guided DNA endonuclease enzyme, and guide RNA (gRNA), a short nucleotide (20 nucleotides) that has a complementary sequence to the target double-stranded DNA (ssDNA) [41]. CRISPR-Cas9 plays a role in novel genome editing tools and has revolutionized the field of genetic engineering in biomedical sciences, clinical research, biotechnology, and agriculture [42,43]. Currently, CRISPR-Cas9 has been developed as a simple and highly sensitive platform for the detection of nucleic acids. The combination of CRISPR-Cas9 with strand displacement amplification (SDA), an isothermal amplification technique, has been reported as a method for ultrasensitive DNA detection [44]. CRISPR-Cas9-triggered nicking endonuclease-mediated strand displacement amplification (CRISDA) has been used for double-stranded DNA detection with high sensitivity at the attomolar level. Furthermore, this technique can be used to detect single nucleotides in the target DNA.

CRISPR-Cas9 has been used as a detection method for miRNAs with low cost, high specificity, and sensitivity [45]. The novel technique, called the RCA-CRISPR-split-HRP or RCH technique, is made up of a combination of isothermal amplifications, the CRISPR-Cas9 as a nucleic acid detection method and split-horseradish peroxidase (HRP) as a reporting method. First, the target miRNAs were bound specifically with a dumbbell probe and amplified using rolling circle amplification with strand displacement DNA polymerase phi29. For the detection step, dCas9 protein, part of the CRISPR-Cas9 system, was fused with split-HRP reporting sequences and bound to RCA products using specific single-guide RNAs (sgRNAs). HRP activity in dCas9 was detected using 3,3′,5,5′-tetramethylbenzidine (TMB). miRNA can be detected within 4 h with high sensitivity at femtomolar concentrations.

CRISPR-associated protein (Cas) 13 is an RNA-guided RNase composed of four members: Cas13a, Cas13b, Cas13c, and Cas13d [46]. The structure of Cas13a is composed of two higher eukaryotes and a prokaryote nucleotide-binding domain (HEPN) with CRISPR-RNA (crRNA)-guided single-stranded RNA cleavage activity [47]. In 2016, the collateral cleavage activity, RNase activity that cleaves non-specific RNA surrounding the target after the binding of Cas13, was also discovered. Cas13 was developed as a platform for nucleic acid detection with high sensitivity.

Specific high-sensitivity enzymatic reporter unlocking (SHERLOCK), a CRISPR-based platform for the ultrasensitive detection of DNA or RNA, was first established in 2017 by Zhang et al. SHERLOCK is the technique that combines the nucleic acid pre-amplification method, which is an isothermal-based amplification, and CRISPR-associated protein 13 (Cas13)-based detection, which can bind and cut the sequences through endonucleolytic activity [48]. 

**Figure 5:**
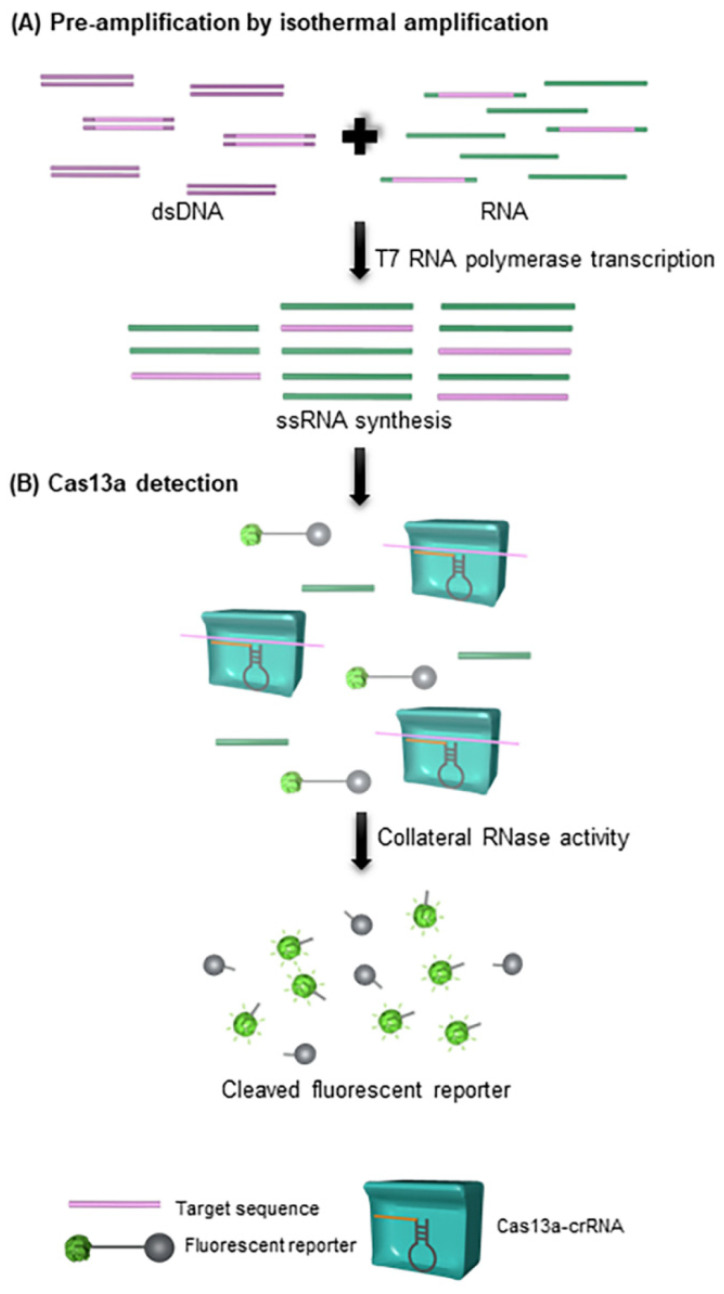
Schematic of SHERLOCK mechanism (redrawn according to [48]).

Figure 5 shows a schematic of the SHERLOCK mechanism. In principle, target DNA or RNA is amplified under isothermal amplification using the recombinase polymerase amplification (RPA) technique. The products were converted to single-stranded RNA (ssRNA) by T7 RNA polymerase transcription reaction. Subsequently, the target sequences of ssRNA were captured by the guide RNA (gRNA) in the CRISPR-Cas 13 complex, which is a complex of RNA sequences made up of two components, including CRISPR-RNA (crRNA), a short nucleotide sequence (17–20 nucleotide) complementary to the target sequence, and trans-activating crRNA (tracr-RNA). After the binding of gRNA to the target sequence, the Cas13 endonuclease was activated, which cleaved the target sequence and triggered promiscuous RNase activity to cleave the surrounding ssRNA. For detection, fluorescently labeled reporter-conjugated ssRNA was added and quenched when promiscuous RNase activity was activated [49]. In 2017, this technique was used to detect Zika (ZIKV) and Dengue (DENV) viral RNA [48]. SHERLOCK can distinguish between ZIKV and DENV and detect the viral particles at concentrations as low as 2 aM, and ZIKV can be detected in clinical specimens where the titer concentrations are as low as 3.2 aM or 2 × 10^3^ copies/mL. In addition, SHERLOCK was able to detect single nucleotide polymorphisms (SNPs) and mutations in cell-free DNA (cfDNA). However, this technique has some limitations. The system is not user-friendly, requires a fluorescence detector for signal readout, and the results are not quantitative.

The second version of SHERLOCK has been developed to overcome these limitations and is called SHERLOCK version 2 or SHERLOCKv2. The new version of SHERLOCK was further developed under four conditions: a multiplexed detection platform for four targets, quantitative analysis, increasing signal sensitivity, and integration with the lateral flow for easy readout [50]. After the improvement, SHERLOCKv2 was tested to detect ZIKV and DENV ssRNA, and it was found that the ssRNA of viruses was detected within 90 min with a sensitivity as low as 2 aM.

CRISPR-associated protein (Cas) 12, an RNA-guided DNase enzyme, functions as a prokaryotic defense mechanism and comprises diverse family members [51]. The structure of the CRISPR-Cas12 system, especially Cas12a, is composed of two parts: CRISPR-RNA (crRNA) with T-rich protospacer adjacent motif (PAM) sequences that guide double-stranded DNA (dsDNA) to cleave at the active site of the catalytic domain and a single RuvC endonuclease domain for dsDNA cleavage [52]. Similar to Cas13a, Cas12a exhibits collateral cleavage activity that cleaves non-specific single-stranded DNA surrounding the target [53]. CRISPR-Cas12a has recently emerged as a novel platform with rapid and high sensitivity for nucleic acid detection.

DNA endonuclease-targeted CRISPR trans-reporter, or *DETECTR*, is a sensitive DNA detection platform using CRISPR-Cas12a, developed in 2018 by Doudna et al. DETECTR is a one-pot DNA detection method that couples isothermal amplification, enhancing the sensitivity, and Cas12-based detection [53]. In principle, the target DNA was amplified at a constant temperature using the RPA assay, and the products were used in the Cas12a reaction. Unlike Cas13a, Cas12a can bind directly to the target DNA using gRNAs with complementary sequences. Next, the formation of a complex of target DNA/gRNA/Cas12a triggers target DNA cleavage via Cas12a endonuclease activity and collateral activity. DETECTR was used to detect dsDNA in HPV with high specificity and sensitivity. This technique can identify and distinguish HPV types 16 and 18 in patient samples within 1 h, and it exhibits high sensitivity at attomolar levels [53]. Furthermore, the DETECTR technique coupled with a fluorescent-biotin reporter has been used to directly detect HPV DNA in liquid samples without DNA isolation. Moreover, the CRISPR-Cas12a detection platform was integrated with the lateral flow technique. The limit of detection of this technique was 0.24 fM compared to detection by PCR-based assay [54].

During the pandemic of SARS-CoV-2, or COVID-19, the DETECTR technique, coupled with lateral flow technology, was developed as a rapid detection platform for SARS-CoV-2 viral nucleic acids [55]. RNA was extracted from nasopharyngeal or oropharyngeal swabs and amplified using primers targeting nucleoprotein (N) and envelope (E) genes using the RT-LAMP technique. Then, DETECTR was performed using RT-LAMP products, and a lateral flow strip was used as a readout technique. This technique can detect 10 copies/μL of nucleic acid of SARS-CoV-2 in 30 min and does not cross-react with related strains of coronavirus. However, CRISPR-Cas12-based detection techniques require two separate steps: (1) pre-amplification of nucleic acid using an isothermal amplification-based technique and (2) CRISPR-Cas-based detection, which is composed of multiple steps and allows sample contamination. A one-pot CRISPR-Cas-based detection technique has been developed to overcome these limitations. 

All-In-One Dual CRISPR-Cas12a or AIOD-CRISPR, a novel CRISPR-Cas-based detection platform with rapid, high sensitivity, and specificity for nucleic acids was developed by Liu et al. [56]. In this system, a pair of Cas12a-crRNAs were used to bind two corresponding sites of the target sequences, and the reaction was mixed and performed in a single tube. First, isothermal amplification was initiated, and then the Cas12a-crRNA complexes were bound to the target site of the amplified products. The products were captured by Cas12a-crRNA and collateral cleavage activity was activated. Similarly, the binding of Cas12a-crRNA to the target site triggers Cas12a endonuclease activity and cleaves fluorescence reporters to generate fluorescence signals. This technique can detect synthetic HIV-1 DNA as low as 1.2 copies in 40 min and can detect 11 copies of HIV-RNA when coupled with reverse transcription techniques. The AIOD-CRISPR system has also been used to detect the nucleic acid of SARS-CoV-2. This technique can be detected 1.3 copies/μL of the synthetic N gene DNA of SARS-CoV-2 in 40 min without any cross-reaction to SARS and MERS coronaviruses. 

To lower the LOD, many published CRISPR biosensing methods require nucleic acid amplification prior to CRISPR sensing. Among biosensing strategies, CRISPR-Cas systems exhibit high sensitivity and specificity and are simple and rapid. The trans-cleavage activity of Cas12 effector proteins is considered a novel potential biosensing application because of its shorter reaction time, high sensitivity, specificity, programmability, and universal applicability. To achieve an ultrasensitive CRISPR sensing platform, combining nucleic acid amplification strategies is often necessary, particularly isothermal amplification.

CRISPR/Cas combining loop-mediated isothermal amplification, namely CIA, was developed in 2020 by Mukama and his colleagues [57]. They integrated the trans-cleavage activity of Cas12a with LAMP amplification to develop an ultrasensitive and specific lateral flow biosensor (LFB) for the detection of *Pseudomonas aeruginosa*. The principle of CIA is based on LFB detection of a biotinylated ssDNA reporter. Samples were amplified by LAMP to improve assay sensitivity. The CRISPR-Cas12 reaction was subsequently demonstrated to enhance the trans-cleavage of Cas12 to cleave the specific target and ssDNA reporter. Reporter cleavage was captured by gold nanoparticle-streptavidin (AuNP-SA) to form the AuNP-SA-biotin ssDNA reporter complex. The complex was captured by a DNA probe, which is a DNA sequence complementary to the reporter, and immobilized at the test line. The excess complex subsequently interacted with biotinylated rabbit polyclonal anti-IgG antibody in the control line. The concept of this approach is that in the presence of target DNA, a trans-cleaved ssDNA reporter cannot bind to a complementary DNA probe immobilized on the LFB test line, resulting in the absence of the test line.

The presence of a test line indicates the absence of a target. The performance of CIA-based LFB exhibits ultrasensitive detection by detecting as low as a single copy-cloned *P. aeruginosa* acyltransferase gene, 1 cfu/mL plasmid containing *E. coli* DH5α pure cultures. This assay did not show cross-reactivity with other nontarget bacteria. The result can be read by the naked eye after 15 min of LAMP amplification, 30 min of Cas12 reaction, and 5 min of LFB readout.

**Figure 6:**
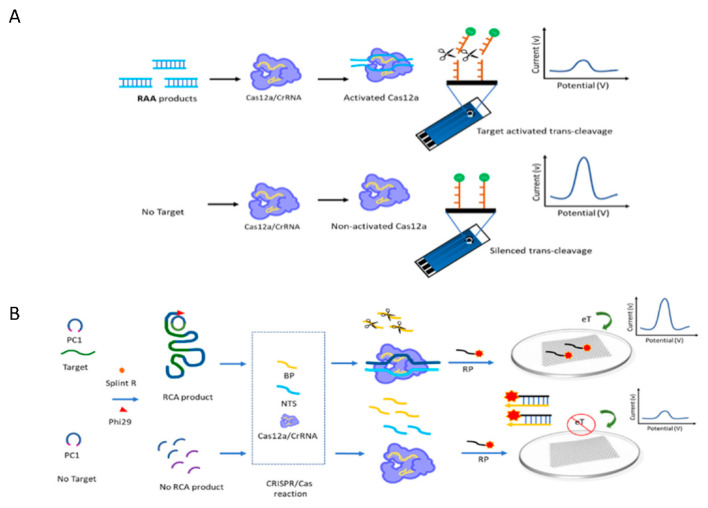
An overview of CRISPR/Cas biosensor combined with the isothermal amplification method described in the literature. (**A**) RAA-based E-CRISPR [59] and (**B**) RCA-CRISPR/Cas12a system [60].

An alternative method, the CRISPR/Cas 12a based electrochemical platform (E-CRISPR), was developed by Dai and co-workers in 2009 for the detection of bacterial DNA [58]. They introduced a DNA target into a disposable screen-printed carbon electrode (SPCE) sensor. The trans-cleavage activity of Cas12a was investigated using an ssDNA reporter linked with methylene blue (MB), an electrochemical tag, on a sensor surface. Trans-cleavage activity is activated in the presence of the target, leading to the cleavage of the MB-ssDNA reporter off the electrode surface. The MB-ssDNA reporter was subsequently released from the electrode, resulting in a decrease in the MB signal transduction. Conversely, in the presence of a cognate target, the MB-ssDNA reporter sensor is retained on the surface. Therefore, an increase in MB transduction was observed. Based on the concept of E-CRISPR, Fan Li and colleagues utilized the potential of recombinase-assisted amplification (RAA) to improve the sensitivity of E-CRISPR, namely RAA-based E-CRISPR [59] for the detection of *Listeria monocytogenes* with the LOD as low as 0.68 aM (genomic DNA) and 26 cfu/mL (pure cultures). The concept of RAA-based E-CRISPR is illustrated in Figure 6A.

The combination of CRISPR-Cas12a with rolling circle amplification (RCA) has been demonstrated for the detection of miRNAs by Min Qing et al., who also integrated this system with an immobilization-free electrochemical biosensing platform [60]. First, the target RNA was amplified by the RCA reaction to obtain a long concatemer DNA sequence. Then, they utilized the trans-cleavage of Cas12a to cleave the universal blocker probe (BP) as the trans-cleavage substrate for the Cas12a enzyme. Later, a universal reporter probe (RP) labeled with MB as an electroactive substance was designed to hybridize with BP. In the presence of a cognate target, the activated Cas12a enzyme can digest BP, resulting in free single-stranded RP labeling with MB tightly adsorbed on the reduced graphene oxide (rGO) electrode, leading to an obvious increase in the electrochemical signal. A promising strategy can achieve highly sensitive and specific detection of miRNAs, parvovirus B19 DNA, and adenosine-5′-triphosphate at 0.83 aM, 0.52 aM, and 0.46 pM, respectively. The concept of the RCA-CRISPR/Cas12a system is illustrated in Figure 6B.

## 5. Conclusions

IA is a powerful polymerization technique with high specificity and sensitivity for disease diagnosis. IA is an efficient target nucleic acid amplification strategy, which is resilient and flexible in diverse approaches. The rational pre-design of probe sequences (for example, encoded sequences of padlock probes in the RCA reaction) can provide multiple functions. IA can be used to detect genomes, proteins, and single cells. In the biomedical field, compared with conventional methods, IA is less time-consuming and less complex in terms of operation. Therefore, IA can be a highly efficient approach for rapid point-of-care diagnostic techniques. All the current applications of the isothermal amplification technologies and the relevant references are shown in Table 1.

## 6. Perspectives

Importance: Although PCR is widely used in the general healthcare system, it is rarely used in resource-limited hospitals because of the requirement of skilled staff and thermocycle controller systems. Therefore, IA, which is a technology that amplifies nucleic acids using a simple thermal controller system and requires only less skilled staff to perform the test, is potentially applied for onsite testing in remote hospital/healthcare. 

Current understanding and challenges: Three popular IA techniques, the LAMP, RCA, and CRISPR systems, have been continuously developed for disease diagnosis. Among these three IA systems, LAMP has been widely used for POC applications because of the short amplification times. However, there are two big challenges waiting for improvement, which are non-specific amplification and primer-dimer formation. For the RCA, it is notable for highly sensitive and selective detection. In particular, the ability to perform the reaction at a low temperature without the requirement of an initial denaturation. However, optimally designed circular DNA or RNA templates are required. Considering the CRISPR-Cas systems, they are high sensitivity and specificity diagnosis platforms, but their operation times are defective. As one IA technique is not suitable for all target molecules and sample types, optimization and clinical evaluation are required to achieve QC/FDA authorization. 

Future direction: Different biomarkers and sample types are somewhat affected, either by enzymes in the IA system or sample preparation, leading to only a few IA techniques being used for disease detection. Therefore, optimization of sample preparation and enzyme engineering to obtain specific binding and special activity in each individual IA system is required. To push new IA technologies into the market, strategic communication on this topic with the QC/FDA regulation sector is also necessary. 

## Figures and Tables

**Table 1:** A table summarizing all the current applications of the isothermal amplification technologies and the relevant references.

Isothermal Amplification Technique	Application	Target	Detection	References
Loop-mediated isothermal amplification (LAMP)	Detection of animal pathogen DNA	Koi Herpesvirus (KHV) DNA	Fluorescent dyes	[3]
	Detection of human pathogen DNA	*Leptospira* DNA	Fluorescent labeled probes	[4]
	Detection of human pathogen DNA	*Mycobacterium spp* DNA	Fluorescent dyes	[5]
	Detection of viral RNA	Middle East Respiratory Syndrome-Coronavirus (MERS-CoV)	Fluorescent dyes	[6,7]
	Detection of viral RNA	Severe Acute Respiratory Syndrome-related coronavirus (SARS-CoV)	Fluorescent dyes	[7]
	Detection of viral RNA	Novel coronavirus (COVID-19)	Fluorescent dyes	[8,9]
Rolling circle amplification (RCA)	Detection of food-borne pathogens	*Listeria monocytogenase* DNA	Fluorescent dyes	[19]
	Detection of pathogen DNA	*Mycobacterium* spp. genomic DNA	Fluorescent labeled probes	[32]
	Detection of viral RNA	Influenza virus (H5N1)	Fluorescent dyes	[33]
	Detection of viral RNA	Severe Acute Respiratory Syndrome-related coronavirus (SARS-CoV)	Fluorescent dyes	[34]
	Detection of miRNAs	miRNA in serum	Fluorescent labeled probes	[35]
	Detection of cancer biomarker	miRNA in serum	Fluorescent labeled probes	[36]
CRISPR-Cas system	Detection of DNA	Human cell lines (HEK293) and human cancer cell lines (MCF-7)	Fluorescent labeled probes	[44]
	Detection of miRNAs	miRNA in serum from cancer patient	Colorimetric	[45]
	Detection of viral RNA	Zika virus (ZIKV) and Dengue virus (DENV)	Fluorescent labeled probes	[48]
	Detection of viral DNA	Human papillomavirus (HPV)	Fluorescent labeled probes	[53]
	Detection of viral RNA	Novel coronavirus (COVID-19)	CRISPR/Cas-LAMP lateral flow	[55]
	Detection of bacterial DNA	*Pseudomonas aeruginosa*	CRISPR/Cas-LAMP	[57]
	Detection of bacterial DNA	*Listeria monocytogenes*	Electrochemical biosensor	[58]

## Abbreviations

AuNPs, gold nanoparticles; CIA, CRISPR/Cas combined loop-mediated isothermal amplification; DETECTR, DNA endonuclease-targeted CRISPR trans reporter; HRCA, hyperbranched rolling circle amplification; IA, isothermal amplification; LAMP, loop-mediated isothermal amplification; LFB, lateral flow biosensor; NAAT, nucleic acid amplification technology; RAA, recombinase-assisted amplification; RCA, rolling circle amplification; SDA, strand displacement amplification; SHERLOCK, specific high-sensitivity enzymatic reporter unlocking.
